# Finger extensor variability in TMS parameters among chronic stroke patients

**DOI:** 10.1186/1743-0003-2-10

**Published:** 2005-05-31

**Authors:** Andrew J Butler, Shannon Kahn, Steven L Wolf, Paul Weiss

**Affiliations:** 1 Departments of Rehabilitation Medicine, Emory University School of Medicine, Emory University, Atlanta, USA 30322, GA; 2 Medicine, Emory University School of Medicine, Emory University, Atlanta, USA 30322, GA; 3 Cell Biology, Emory University School of Medicine, Emory University, Atlanta, USA 30322, GA; 4 Department of Psychology, Emory College, Emory University, Atlanta, USA 30322, GA; 5 Department of Biostatistics, Rollins School of Public Health, Emory University, Atlanta, USA 30322, GA

**Keywords:** motor mapping, reliability, center of gravity, upper limb, plasticity, rehabilitation, cortex

## Abstract

**Background:**

This study determined the reliability of topographic motor cortical maps and MEP characteristics in the extensor digitorum communis (EDC) evoked by single-pulse TMS among patients with chronic stroke.

**Methods:**

Each of ten patients was studied on three occasions. Measures included location of the EDC hotspot and center of gravity (COG), threshold of activation and average amplitude of the hotspot, number of active sites, map volume, and recruitment curve (RC) slope.

**Results:**

Consistent intrahemispheric measurements were obtained for the three TMS mapping sessions for all measured variables. No statistically significant difference was observed between hemispheres for the number of active sites, COG distance or the RC slope. The magnitude and range of COG movement between sessions were similar to those reported previously with this muscle in able-bodied individuals. The average COG movement over three sessions in both hemispheres was 0.90 cm. The average COG movement in the affected hemisphere was 1.13 (± 0.08) cm, and 0.68 (± 0.04) cm) for the less affected hemisphere. However, significant interhemispheric variability was seen for the average MEP amplitude, normalized map volume, and resting motor threshold.

**Conclusion:**

The physiologic variability in some TMS measurements of EDC suggest that interpretation of TMS mapping data derived from hemiparetic patients in the chronic stage following stroke should be undertaken cautiously. Irrespective of the muscle, potential causes of variability should be resolved to accurately assess the impact of pharmacological or physical interventions on cortical organization as measured by TMS among patients with stroke.

## Background

Single pulse Transcranial Magnetic Stimulation (TMS) is a safe and noninvasive technique for mapping cortical motor representation [1-4]. Recently, TMS has been used to explore mechanisms underlying both spontaneous and therapy-induced post-stroke motor recovery. In this context, most interventional studies have not considered intra-subject variability of TMS maps prior to the provision of a therapy, thus implying that cortical changes are attributable to the intervention. However, our laboratory recently demonstrated significant variability within able-bodied, right hand dominant participants across sessions and between hemispheres, for distance between the lowest resting motor threshold locations for a muscle (hotspot), center of gravity distance, and normalized map volume TMS parameters when mapping the extensor digitorum communis (EDC) muscle [5]. Adjusting for time and examining mean changes for hemispheres across sessions revealed that there was a 9-fold greater movement over sessions in the left hemisphere among these variables. Previous studies have shown reproducible motor maps of abductor pollicis brevis (APB) and abductor digiti minimi (ADM) [6] in both healthy subjects [6] and chronic stroke patients [7] using conventional electrode placement. In addition, Wasserman et al. (2002) found no systematic changes in resting and active motor evoked potential (MEP) thresholds among 19 women across three sessions.

However, few studies have examined the inherent variability in TMS motor maps in chronic stroke subjects not receiving an intervention. This preliminary study represents one of the first efforts to evaluate intra-subject variability in TMS motor maps of chronic stroke patients during three separate mapping sessions. As in a previous report on able-bodied participants [5], we chose to map EDC because this muscle is often affected by a stroke and its volitional activation is important in overcoming the profound flexion posture at the hand and wrist that characterizes many patients. Furthermore, the EDC is near the skin surface, making it a convenient and more precise site for electromyography recording due to its close proximity to other finger and wrist extensors which limits effects of cross talk, undesired overflow effects and, if present, volume-conducted pick up by muscles with comparable function.

Therefore, the present study is unique because of the specificity of recording using closely spaced electrodes and the repetitive sessions permitting examination of variability in TMS-related measures for the EDC muscle in patients greater than two years post stroke. The inherent variability seen in TMS measures following physical or pharmacological interventions would need to be less than that seen under non-interventional conditions to be assured that changes induced by these interventions are associated with cortical reorganization.

## Methods

### Design

This study used repeated measures, non-random sampling design. Motor maps for the EDC were created for each hemisphere during all three sessions for every subject. Sessions were separated by approximately seven days.

### Chronic stroke patients

Ten right-handed patients who suffered a stroke greater than 2 years prior to testing were recruited using consecutive sampling of all chronic stroke patients who had the ability to extend ≥ 20° at the wrist and 10° at the fingers [8]. Specific upper extremity motor deficits were similar to those seen in patients enrolled in a multisite randomized trial to investigate the effect of constraint-induced movement therapy in improving upper extremity function among adults recovering from a cerebrovascular stroke [9]. The medical condition of each patient was stable. Each volunteer was living independently within the community and ambulated independently. For this preliminary study, patients with a wide range of cortical lesions and chronicity were studied. Basic information about age, gender, hand dominance, time since stroke and lesion site is found in Table 1. Four of ten patients had strokes that primarily affected their non-dominant upper extremity. Data from nine able-bodied volunteers collected in a previously reported TMS variability study were used as a comparison group [5].

**Table 1:** Clinical data for patient volunteers.

**Participant**	**Age**	**Gender**	**Hand Dom.**	**Months since Stroke**	**Site of Lesion**
1	58	Male	R	32	Left Lacunar Infarct CVA
2	55	Male	R	34	Left thalamic ICH and right subcortical lacunae
3	78	Male	R	35	Right Internal capsule lacunar CVA
4	56	Female	R	56	Right cerebral hemisphere
5	46	Female	R	54	Right putamen hemorrhage
6	70	Female	R	98	Right cerebral hemisphere
7	60	Female	R	147	Left cerebral hemisphere
8	56	Male	R	85	Left cerebral hemisphere
9	56	Male	R	33	Left lacunar infarct corona radiate
10	67	Female	R	25	Left cerebellum

Participants were excluded if they had: a history of epilepsy, psychiatric disorders, fracture in the upper extremity within the past two years, diaphoresis, severe spasticity, tendonitis in the upper extremity within the last three months, migraine headaches within the last six months, Attention Deficit Disorder, or Attention Deficit Hyperactivity Disorder. In addition, participants could not be receiving stimulant or relaxant medications, (including anti-spasticity medication or pharmacological injections) demonstrate current exacerbation of osteoarthritis in the upper extremity or of rheumatic disorders, or be participating in sports that require excessive wrist extension for more than once per week over the previous three months. Volunteers read and signed an informed consent form previously approved by the local University Institutional Review Board.

### Measurements/Instrumentation

Details about the experimental design and data collection methods have been presented previously [5]. Briefly, the following variables were measured at each session: hotspot and active site locations, hotspot excitability threshold, average MEP amplitude for hotspot and active sites, and recruitment curve slope. The hotspot was defined as the grid location where the motor threshold was the lowest while evoking the largest response [10]. Given the comparatively closer inter-electrode recording distances, active sites were designated as the grid locations where a response of ≥ 25 μV in 5 out of 10 trials at 110 percent of resting motor threshold was obtained. Each site with five consecutive responses less than 25 μV was considered non-active. Mapping was complete when locations adjacent to the active sites were identified as non-active. Recruitment curves were generated to evaluate the relationship between MEP amplitudes at the hotspot and progressively increasing stimulus intensities until the curve flattened. The slope of the recruitment curve is thought to be a function of the physical distribution of stimulus excitation from the coil and yields a measure of distribution of the excitability in the cortex [11].

The average MEP amplitude for the hotspot, center of gravity (COG), normalized map volume, and slope of the recruitment curve, were calculated following data collection. COG was defined as the map location representing the amplitude-weighted center of the area of excitability [12]. Normalized map volume was defined as the area of the map multiplied by the normalized MEP amplitudes.

Normalization of mean amplitudes (nMEP) was completed for all coordinates for each participant by dividing the mean amplitudes by the maximum mean amplitude. The normalized map volume (nMV) was calculated by adding all of the nMEP amplitudes and multiplying by the area [13]. The X and Y coordinates for each active site were multiplied by the normalized MEP amplitude (X*nMEP and Y*nMEP), and the sum of all the values was calculated respectively. The center of gravity (COG) X coordinate was calculated by  and COG Y coordinate was calculated by [12].

The recruitment curve (RC) was generated by examining MEP amplitudes at the hotspot over progressively increasing intensities, thus providing information about cortical excitability. This was done by placing the coil at the hotspot and recording 5 stimuli in 10% increments beginning at an intensity of 10 % below threshold. Data collection for the RC was terminated when a plateau of the sigmoidal curve was observed. When calculating the RC slope, the first two data points collected were omitted because they were at sub-threshold levels, and the end point of the recruitment curve was determined to be either at 80% stimulator output, where a supra-threshold motor response was observed, or once a plateau in the recruitment curve was noted. The slope of the recruitment curve was generated from the resultant data points using linear regression.

The MEPs were recorded using two 7 mm × 4 mm silver-silver chloride surface electrodes (Medtronic, Inc., Minneapolis, MN) separated by approximately 1.5 centimeters. The peak-to-peak amplitude of the unrectified MEP was measured automatically using custom established routines created in LabView 6.0 (National Instruments, Austin, TX) in each of the 10 trials in each block, and their average was calculated for each stimulus site to give the mean peak-to-peak amplitude.

### Reliability

The reliability of data acquisition was assessed by two investigators. One investigator performed the stimulation, while the other monitored the recordings for all sessions. Each investigator performed the same duties throughout the study to decrease the chance of experimenter variability [5]. Potential participants were screened using an inclusion/exclusion criteria questionnaire. To ensure consistent electrode placement for all sessions, the EDC muscle belly was isolated by palpation and then marked at the first session. A clear acetate sheet was applied to each forearm. Marks were then placed on the acetate sheet for electrode placement and relevant anatomical landmarks to assure consistent placement during subsequent sessions. To maintain consistent cap placement across sessions, detailed distance recordings were made from the nasion, inion, and bilateral pre-tragus to the vertex.

## Procedure

### Patient preparation

After isolating each EDC with the wrist in flexion to determine optimal placement of the electrodes, the skin surface over the EDC on the forearms was shaved and abraded with alcohol until erythemic responses appeared. Recording electrodes were placed on the skin over the EDC muscle bellies, and a reference electrode was applied ipsilaterally and proximally to the recording electrodes to reduce EMG noise levels. Skin impedance between active electrodes and between each active electrode and the reference were kept below 2 kilo-ohms (kΩ), and below 20 kΩ respectively.

Each participant was seated in a relaxed position with pillows placed under the forearms and hands. A firm-fitting cap upon which 1 cm^2 ^grids had been imprinted was placed on the participant's head and secured appropriately to serve as a reference for reproducible coil placement and orientation.

### Data collection

EMG data were measured bilaterally through surface electrode pairs, but responses to cortical stimulation were only recorded from the electrodes contralateral to the hemisphere being stimulated. Surface EMG signals were amplified and filtered with an Isolated Bioelectric Amplifier (James Long, Caroga Lake, NY), with bandpass filter settings of 30 and 1000 Hz, and digitally sampled at 1 KHz. 100 ms of prestimulation activity and 200 ms of post-stimulation activity were recorded. Trials in which active contraction contaminated the MEP were omitted, and the trial was repeated. To facilitate subject alertness throughout data collection, the investigator monitoring recordings engaged in neutral conversation with each volunteer between blocks of presentations of stimuli.

Stimulation of each hemisphere at the motor cortex using a 9 cm diameter figure-8 coil MAGSTIM 200 (Magstim Company Ltd., Whitland, Dyfed, UK) was performed in a systematic fashion at 0.2 Hz. The coil was oriented with the handle facing backward so the induced current in the brain was in the posterior-anterior direction during the rising phase of the monophasic pulse. Approximately 300–400 stimuli were delivered in sequential order during the mapping procedure.

Potential hotspot sites were identified using a stimulus intensity that evoked MEPs ≥ 25 μV, in five out of ten trials. Once these cortical sites were identified, the intensity was reduced until the hotspot and the hotspot's excitability threshold for the EDC were determined. Thereafter, the stimulus intensity was increased by ten percent and cortical sites beginning at the hotspot were stimulated to identify the active sites. Mapping was complete when all surrounding inactive sites were identified.

### Data Analysis

The assumption of sphericity was ensured using the Greenhouse-Geisser correction. A two-way repeated measures analysis of variance (ANOVA) was used to explore the difference between sessions, hemispheres, lesion location and the interaction within participants for the following variables: resting motor threshold, map area, mean peak-to-peak MEP amplitude for the hotspot, normalized map volume, slope of recruitment curve, COG centroid and COG distance A and B. For all tests the alpha level was set at α = 0.05. The Euclidean equation was applied to determine the distance the hotspot and COG locations traveled from sessions: one to two (distance A) and two to three (distance B).

To allow for comparison between sessions in a single hemisphere, a centroid point, *Xc, Yc*, was calculated from the three x-and y-co-ordinates for the COG and hotspot positions. The x and y co-ordinates represent the medial-lateral and anterior-posterior distance (cm) from an arbitrary origin (0,0).

## Results

**Figure 1:**
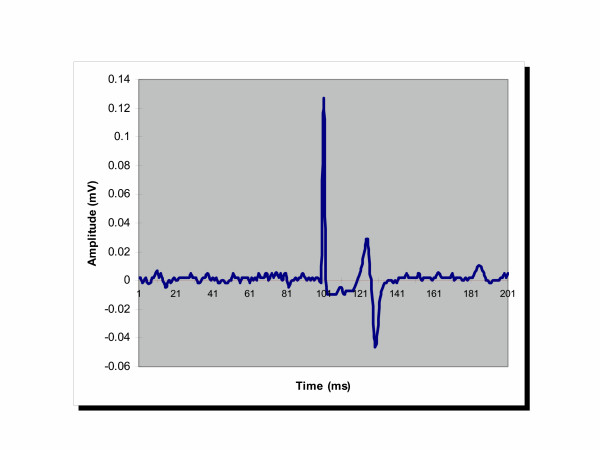
A representative MEP amplitude of 70 μV beginning approximately 20 ms after the stimulus artifact.

The scalp overlying the motor cortex was stimulated at 110% of motor threshold, while recording from EDC. A representative MEP amplitude of 60 μV beginning approximately 20 ms after the stimulus artifact is depicted in Figure 1.

Patient data for the affected and less affected hemispheres are provided in Table 2 (see Additional file 1). The resting motor threshold (RMT) in the affected hemisphere had a minimum value of 43% (case #2, session 3) and maximum value of 100% (case #1, session 3). The RMT values in the less affected hemisphere ranged from 31% (case #4, session 3) to 63% (case #8, session 1).

Map volume in the affected hemisphere ranged from 3.13 cm^2 ^(case #2, session 3) to 13.26 cm^2 ^(case #1, session 1), while in the less affected hemisphere values ranged from 0.038 cm^2 ^(case #6, session 3) to 0.385 cm^2 ^(case #3, session 1) respectively. The minimum MEP amplitude in the affected hemisphere was observed in case #1, session 2 (0.0122 μV) while the maximum 0.1828 μV was observed in case #2, session 1.

The number of active sites in the affected hemisphere ranged from 0 (case #1, session 2) to 19 (case #10, session 2). While the range in the less affected hemisphere was from 3 active sites (case #7, session 3) to 11 (case #1,2,5). Collectively these data would appear to illustrate a substantial degree of variability in all values among these 10 patients with stroke.

**Figure 2:**
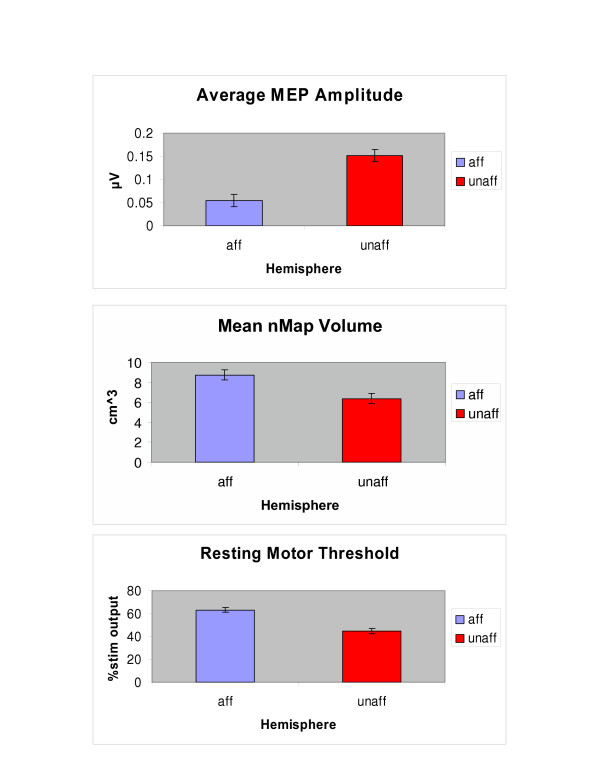
Inter-hemispheric variability collapsed across the three mapping sessions for the parameters: average MEP amplitude, normalized map volume, and resting motor threshold. P-values are depicted in the lower right corner of each plot.

However, analysis of variance showed no between session variability for any of the measured parameters (Table 3). There were no statistically significant interhemispheric (between hemispheres) difference in the number of active sites (F_1,7 _= 0.28; p= 0.6157), and RC slope (F_1,7 _= 3.34 ; p = 0.1106). In contrast, greater interhemispheric variability was observed for: average MEP amplitude (F_1,6 _= 85.01; p < 0.0001), normalized map volume (F_1,7 _= 5.98; p = 0.044), and resting motor threshold (F_1,8 _= 12.79; p = 0.0072) (Table 3). As shown in Figure 2, resting motor threshold was larger for the affected 63.1% (2.1) than the less affected 44.7% (2.1) hemisphere. Normalized map volume was also larger for the affected 8.7 cm (0.5) compared to the less affected 6.3 cm (0.5) hemisphere. Larger MEP amplitudes were recorded in the less affected hemisphere compared to the more affected hemisphere [(0.15 μV (± 0.01) and 0.05 μV (± 0.01)].

**Table 3:** Analysis of Variance for Dependent Variables

	**Hemisphere**		**Session**		**Interaction**	
	F value	P value	F value	P value	F value	P value
			
Motor Threshold	12.79	0.0072*	0.47	0.6336	1.25	0.3139
Average MEP Amplitude	85.01	0.0001*	1.50	0.2628	2.78	0.1016
# Active Sites	0.28	0.6157	0.52	0.6061	0.29	0.7532
Normalized Map Volume	5.98	0.0444*	0.02	0.9759	1.35	0.2914
COG distance	1.22	0.2833	0.53	0.4781	0.06	0.8165
Recruitment Curve Slope	3.34	0.1106	0.67	0.5264	1.17	0.3380

* Indicates statistically significant value; MEP = Motor Evoked Potential

When considering lesion location as a factor (i.e. cortical vs. subcortical), ANOVA revealed no significant differences in any of the dependent variables measured across hemisphere, session or their interaction.

**Figure 3:**
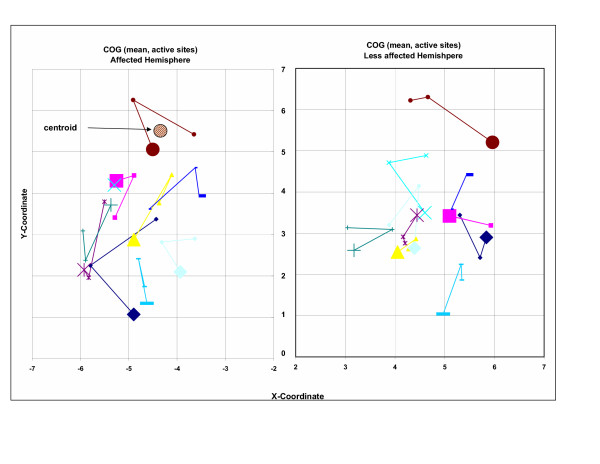
2-D representation of the overall COG movement (cm) across three sessions for each participant and both hemispheres. First session is demarcated by a larger symbol. The COG was calculated using mean MEP amplitudes shown for active sites only. Larger numbers on the x-coordinate and y-coordinates represent lateral and anterior scalp stimulus locations, respectively. Note that locations are unadjusted for the repeated measures on hemisphere and session. Each grid location represents one centimeter. The hatched circle represents the COG centroid location for a single subject in one hemisphere. All centroids are displayed in Figure 4.

The ANOVA comparing COG distances A versus B were not significant between hemispheres or sessions (Table 3). The magnitude and range of COG movement between sessions were similar (Figure 3, Table 4) to those reported in a previous mapping study of this muscle with able-bodied individuals [5]. The average COG movement over three sessions in both hemispheres was 0.90 cm. The average COG movement in the affected hemisphere was 1.13 (± 0.08) cm, and for the less affected hemisphere 0.68 (± 0.04) cm among our stroke participants.

**Table 4:** Average and range of COG movement across session 1 (S1), session 2 (S2) and session 3 (S3) and between hemispheres. SD = standard deviation.

**Hemisphere**	**Mean (cm)**	**SD**	**Range (cm)**
Affected			
S1→S2	1.04	0.55	0.21→ 1.75
S2→S3	1.14	0.54	0.68A1.87
S3→S1	1.20	0.70	0.45A1.70
**Ave**	**1.13**	**0.08**	**0.21→1.87**
Less affected			
S1→S2	0.90	0.41	0.49→ 1.70
S2→S3	0.62	0.39	0.16A0.90
S3→S1	0.53	0.34	0.02A0.91
**Ave**	**0.68**	**0.04**	**0.02→1.70**

**Figure 4:**
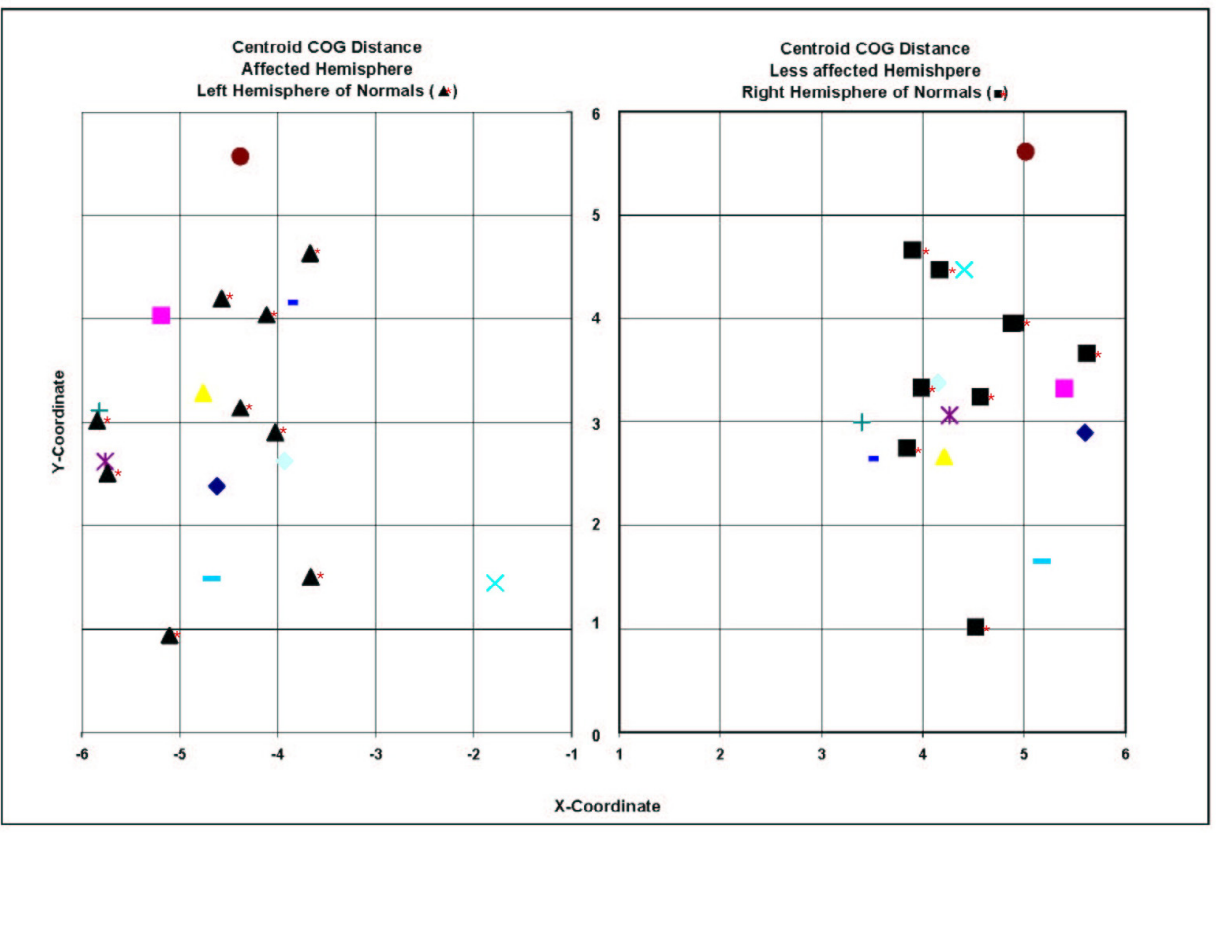
Centroid location of COG for the affected and less affected hemisphere for individual patients along with 9 able-bodied adults. The left hemisphere corresponds to the dominant arm in able-bodied participants and the affected and less affected hemispheres are of mixed hand dominance for the patients No significant variability exists when comparing left or right hemisphere of right handed able-bodied individuals with affected (p = 0.996) and less affected (p = 0.68) hemispheres of patients. Symbols with asterisks (*) represent centroids for left hemisphere (triangle*) and right hemisphere (square*) of able-bodied individuals. Each grid location represents one centimeter.

To allow for comparison between sessions in a single hemisphere, a centroid point was calculated. No significant difference was observed between the affected and less affected hemispheres across three sessions for COG centroid (Figure 4). No significant interhemispheric (between hemisphere) or intrahemispheric (between session) variability was observed for the COG centroids (p = 0.6611).

There were no significant differences in movement of COG centroid between the left or right hemisphere of healthy right handed individuals [5] and the affected (p = 0.996) or less affected (p = 0.68) hemisphere of right handed patients with stroke. All of our able-bodied volunteers were right hand dominant, and all of our patients were right hand dominant. Therefore, both groups could be compared. Figure 4 indicates that the COG centroid location for the affected and less affected hemisphere for individual patients along with 9 able-bodied adults show considerable overlap.

## Discussion

This study demonstrated consistent between session measures for all the recorded variables. Consistent between hemisphere measures were obtained for the number of active sites, COG distance and recruitment curve slope, when recording EDC maps using single pulse TMS among patients greater than 2 years after stroke. In contrast, between hemispheres variability was observed in three measures: the average MEP amplitude, normalized map volume and resting motor threshold.

These findings support previous studies which report reproducible motor maps of the abductor pollicis brevis [6,7,10] and abductor digiti minimi [6] in both healthy subjects [6,10] and chronic stroke patients [7].

### Interhemispheric variability collapsed across the three mapping sessions

Our data are in accord with previous reports on patients with stroke showing that resting motor threshold is significantly higher and MEP amplitudes are smaller in the affected hemisphere compared to the less affected hemisphere and that the relationship is reproducible between sessions [7,14,15].

The larger normalized map volume of EDC in the damaged hemisphere may be due to the dynamic alteration in the pattern of brain activity in response to change in afferent signals, efferent signals and/or adjustment to injury (i.e. neuroplasticity). In the current study, six of ten patients reported strokes that primarily affected their dominant upper extremity. Although behavioral data were not collected prior to TMS mapping, all patients reported living within their communities and using their more impaired upper extremities for many activities of daily living. None of the volunteers were receiving formal training (i.e. constraint induced therapy) at the time of testing, however, they would have met the inclusion criteria to participate in a randomized clinical trial of constraint induced therapy that required initiation of wrist and finger extension [9]. Their repetitive efforts at using the more impaired arm may have contributed to modifying functional reorganization of remaining cortical tissue in the corresponding hemisphere. This use may have consequently led to a comparably larger map size.

Motor or sensory activity in one arm can affect the other arm. There is the potential for input from the ipsilateral (ie. less impaired hand) side to the damaged side of the brain. Frequent use of the less impaired limb may have led to a map volume increase on the ipsilateral (affected hemisphere). There is now evidence that such modulatory effects can occur with practice [16] and has the potential to occur with mild or strong voluntary contractions [17]. Further data collection is necessary to completely explore this theory.

The much greater COG movement across sessions in the damaged hemispheres of stroke patients than in undamaged hemispheres of both stroke patients and comparison group is likely related to greater map volume in the damaged hemispheres. The calculation of COG x- and y-coordinates is dependent upon MEP amplitude (nMEP), and normalized map volume (nMV). The normalized map volume is directly proportional to the number of active sites. Large intersession variation in either of these values will affect the COG value and subsequent calculation of displacement between sessions. Although the variability in MEP amplitude was comparable between hemispheres, closer inspection of the data indicated up to a 58% greater variation in the number of active sites between sessions on the affected hemisphere (mean = 8.13 ± 03.94) compared to the unaffected hemisphere (mean = 8.3 ± 02.30). The increased variability in the number of active sites in the affected hemisphere is a contributing factor to the greater COG movement between sessions observed in the affected hemisphere.

### Overall COG movement across three sessions for each participant and both hemispheres

The center of gravity remained consistent over the three sessions, with the majority of movement occurring in the anterior or posterior directions, along the Y-axis (Figure 3), an observation consistent with the TMS-induced field generated from the figure of eight coil orientation [18]. The average COG movement in the less affected hemisphere, 0.68 (± 0.04) cm is equivalent to the average COG movement 0.68 (± 0.02) cm measured from EDC in nine able bodied adults [5]. The average COG movement in the affected hemisphere reported here is about 60% greater when compared to the less affected hemisphere (Table 4). These changes in COG shift between session and across hemispheres are considerably larger than measures reported by Liepert et al. in a previous study of stroke patients' undergoing an intervention [7]. Their measurement for COG displacement in the abductor pollicis brevis (APB) was 0.234 ± 0.21 cm in the media-lateral axis in the affected hemisphere and 0.153 ± 0.18 cm in the less affected hemisphere and 0.71 ± 0.47 and 0.50 ± 0.426 cm in the anterior-posterior axis for the affected and less affected hemispheres, respectively.

The difference in magnitude may be a function of how COG displacement is determined between sessions. Our calculation of the Euclidean distance is fundamentally different than that described by Liepert et al. [19,20]. Liepert's description of the shift in COG between sessions using displacement is useful because it provides an indication of both distance and directional change along one axis. However, concern should be given to the use of a mean displacement, expressed as the difference between two consecutive x- or y-coordinates without considering the absolute value of the calculation. Failure to consider the overall positive and negative directionality of displacement may have led to artificially lower COG shifts in value than seen in the current study (i.e. if first value is negative and second is of equal value positive). In contrast the resulting Euclidean distance between two points is an absolute value. Calculating the Euclidean distance between two points in a plane using the Pythagorean Theorem allows for the creation of a 2-dimensional displacement vector which can better describe the overall change in location between sessions independent of direction.

The calculation of COG is dependent on MEP characteristics which differ for distal and more proximal muscles. For instance, the MEP thresholds in proximal muscles (i.e. deltoid, biceps brachii) are higher and the responses vary more in amplitude from trial to trial than in distal muscles such as abductor pollicis brevis and flexor carpi radialis [21]. Furthermore, the form and structure of MEPs in proximal muscles is often more complex than in distal muscles. Although not statistically different, larger variation in EDC amplitude from trial to trial could be linked to the observed increases in COG movement between sessions because a single large amplitude MEP can have a significant weighting on the overall mean of 10 samples which is then used for subsequent statistical calculation.

Additionally, McDonnell et al. (2004) have noted sufficiently large variability in MEPs recorded under standard conditions so that no significant differences in their magnitude over time can be revealed by conventional statistical analysis (ANOVA). They suggested that if a change in MEP size is expected as a result of an intervention, the change in magnitude must be large or many trials must be included in the analysis, before significant differences can be demonstrated [22]. Therefore a reproducibly large change in MEP amplitude is necessary for significant movement in COG over sessions. However substantial variability in MEPs over trials may also increase COG movement.

### Centroid of COG in both hemispheres among individual patients and able-bodied adults

The calculation of a centroid permits visualization of a geometric locus for COG among cerebral hemispheres of our stroke and able-bodied participants. One would predict slight variations in cortical representation of the EDC between hemispheres. However, there are no predicable shifts in COG from session1 to session 3. Our data provide evidence that there is relative consistency in chronic stroke patients not receiving an intervention.

### MEP characteristics displaying stability between sessions

In this study we observed large fluctuations in MEP amplitude, even under carefully controlled conditions. A previous study [23] found that regardless of the variation in the MEP amplitude, TMS map positions and areas are remarkably stable, with variations on the order of 1 mm for map position and less than 5% for map area. Likewise, our standard deviation in COG values was very small, with a mean value of 1.1 mm in latitude and 1.3 mm in longitude across subjects. In addition, the standard deviation of mean map area was only 1.1 cm^2 ^(3.0%) across subjects. This high stability in COG has been observed in serial studies of patients with unilateral motor problems, in which the less affected side was stable to within 2–3 mm over periods from weeks to years [24].

In our patients with chronic stroke, the average COG movement across sessions in the less affected hemisphere was comparable to those shown previously in able bodied individuals [5], while COG movement across sessions in the affected hemisphere varied more on average than seen among those individuals (Figure 3 and Table 4). This increased variability may have resulted from our patients sustaining an uncontrolled 'relaxed' state compared to a controlled low-level voluntary contraction at 10% of maximum root-mean-square EMG activity [23]. Nonetheless, the COG variability found in our study was insignificant, producing a reliable measure for each patient's hemispheres for all three mapping sessions.

### MEP characteristics showing high variability

The high trial-to-trial variability of MEP amplitude may be attributed to a number of factors. First, a range of cellular excitability levels in both spinal and upper motoneurons, which, under some circumstances, may bring these cells very close to firing threshold without actually discharging. In the case of the upper motoneuron, inherent excitability levels could allow some neurons to reach their discharge threshold but without the appropriate numbers or sufficient spinal synaptic excitability to temporally or spatially enhance EDC motoneuron discharge. Thus, mapping in the relaxed state is complicated by the variations that may occur in corticospinal excitability but could not be measured by monitoring EMG activity.

A second factor that can contribute to the intrinsic differences in MEP amplitude is variability in the desynchronization of the efferent volley [25]. Spontaneous physiological oscillations in motoneuron excitability at both the cortical and spinal levels are uncontrollable and unobservable factors potentially causing significant fluctuations in response size [10]. Changes in the state of the participant's alertness [26], levels of muscle tonicity, or anticipation of movement-specific factors, such as mental imagery [27,28], may also contribute to intra-trial MEP variability.

A third factor contributing to MEP variability may be stimulus intensity. This study recorded MEPs using stimulus intensities of 110% of motor threshold. When using higher stimulus intensities, as reported in some studies [29] there are more motoneurons activated and, therefore, fewer are available to spontaneously discharge in concert and contribute to the MEP amplitude, thereby affecting variability.

Small alterations in the position of the coil also can produce a source of within-subject variability [30]. Although the experimenter can make every effort to hold the coil in a uniform manner on a given scalp location, the identical spot is probably not stimulated at each session. The figure-8 coil can be rotated slightly, yet be the source of immense change in the area of the cortex being stimulated.

Although stimulation with a figure-of-eight coil is often described as focal; 'focusing' the electromagnetic field is in fact not practical. The maximum field is generated at the point under the intersection of the two wings of a figure of 8 coil; however, a divergent field is created surrounding this point. As a result, the spatial distribution of induced current flow can still be quite large, and the possibility of exciting cells under the wings and even cells located some distance from their intersection exists [1].

These factors can produce substantial variations in results obtained from TMS mapping studies. Although some of these factors are near impossible to control in a mapping session, others, such as coil placement and focal stimulation or the level of subject attentiveness, should be addressed as a precursor to TMS mapping studies designed to exploit variables using this modality to interpret results from specific interventions.

### Electrode Placements

We chose the use of closely spaced surface electrodes to measure motor evoked potentials from the EDC because this placement limits the evoked MEPs to the underlying muscle and its associated movement. Previous TMS studies used widely spaced electrode arrays. For example, the placement of electrodes over the abductor pollicis brevis records a wide range of movements caused by the flexor pollicis brevis, adductor pollicis, opponens pollicis or interossei [31]. When comparing evoked responses using typical montage and closer spaced electrode arrays, we demonstrated larger map volumes with the montage configuration compared to close electrode placement. The summation of MEPs that represent multiple muscles seen by the more widely spaced electrode configuration results in greater MEP amplitudes, map volumes, number of active sites, and steeper recruitment curve slopes; however, there is greater difficulty identifying which muscles contribute to the response with each cortical stimulation and the representative movements they subsume. This consideration is important in TMS studies that relate changes in map attributes to function. For example, in mapping the APB [19], by knowing that the traditional placements also monitor volume conducted responses from muscles with a flexion function, would increased maps be teleologically relevant to an intervention designed to enhance movement in patients with stroke?

### Functional Ramifications

Another unique aspect of our study was the focus on activation of the EDC and extension of the fingers. This movement is important in retraining function among patients with stroke, because hand extensor muscles are typically weak or inactive while muscles with a flexion function are disinhibited. Placement of wide spaced EDC surface electrodes previously [32] may actually record motions that are counterproductive to the benefits inherent in the very therapy being instituted.

## Conclusion

This study is one of the very few to examine variability in TMS responses among a small group of patients with chronic stroke. Even with the use of chronic stroke patients and closely spaced electrodes, similarities to previous studies using able-bodied subjects were found. However, not surprisingly, findings that were significantly different from these prior studies were also observed.

Closely spaced electrode placement is important for properly isolating movements in limb muscles. Therefore, TMS studies using this placement array need to be undertaken to determine if resultant maps replicate those generated from previous studies employing more traditional, wider spaced electrode configurations. The potential causes of variability identified in this study, the precision of electrode recordings, or entirely new analysis methods should be considered in an effort to accurately assess pharmacological or physical interventions and their impact on cortical organization.

## Authors' contributions

AB, SK and SW conceived of the study, participated in its design, participated in the data collection and drafted the manuscript. PW participated in the design of the study and performed the statistical analysis. All authors read and approved the final manuscript.

## Abbreviations

COG = center of gravity; TMS = transcranial magnetic stimulation; EDC= extensor digitorum communis; MEP = motor evoked potential; EMG= electromyography

## Supplementary Material

Additional File 1 Table 2 represents patient data for affected and less affected hemispheres. Click here for file
