# Supplementary material for: Finger extensor variability in TMS parameters among chronic stroke patients
Source: J Neuroeng Rehabil. 2005 May 31;2:10. doi: 10.1186/1743-0003-2-10 (PMC1175099; doi:10.1186/1743-0003-2-10)
Supplement: Additional File 1 — Table 2 represents patient data for affected and less affected hemispheres. [file 1743-0003-2-10-S1.pdf]

Table 2. Patient data for affected and less affected hemispheres.

Affected Hemisphere

Case #	RMT (%) Session			Avg. MEP Amp. (V) (Std. Err.) Session						# AS Session			Map Volume (cm^3) Session		
	**1**	**2**	**3**	**1**		**2**		**3**		**1**	**2**	**3**	**1**	**2**	**3**
1	90	99	100	0.0276	(0.001)	0.0122	(0.001)	0.0151	(0.002)	8	0	1	13.268	10.054	1
2	50	49	43	0.1828	(0.044)	0.0926	(0.039)	0.025	(0.006)	8	6	5	4.68	4.707	3.132
3	55	59	57	0.0578	(0.011)	0.0433	(0.013)	0.0262	(0.005)	4	9	14	7.32	10.345	11.389
4	58	55	66	0.0263	(0.005)	ND	ND	ND	ND	6	ND	ND	8.0462	ND	ND
5	47	48	45	0.0322	(0.004)	0.0379	(0.005)	0.0644	(0.008)	12	6	8	11.626	8.135	9.59
6	70	59	62	0.0327	(0.005)	0.0682	(0.006)	0.0268	(0.004)	6	12	10	7.012	7.341	5.75916
7	68	69	73	0.0202	(0.002)	0.0482	(0.003)	0.0298	(0.002)	6	13	13	14.603	7.696	15.646
8	69	72	74	0.0512	(0.004)	0.0462	(0.004)	0.0307	(0.002)	10	5	11	10.47	7.142	8.11538
9	58	58	60	0.0617	(0.010)	0.0968	(0.036)	0.0448	(0.007)	12	13	8	12.023	8.155	5.576
10	56	55	68	0.0349	(0.015)	0.1836	(0.015)	0.0724	(0.007)	9	19	10	4.215	7.824	8.3455

Less Affected Hemisphere

Case #	RMT (%) Session			Avg. MEP Amp. (V) (Std. Err.) Session						# AS Session			Map Volume (cm^3) Session		
	**1**	**2**	**3**	**1**		**2**		**3**		**1**	**2**	**3**	**1**	**2**	**3**
1	45	46	43	0.0802	(0.019)	0.122	(0.033)	0.189	(0.036)	10	11	6	8.093	6.983	3.64
2	43	45	43	0.334	(0.055)	0.0495	(0.006)	0.109	(0.023)	5	11	10	2.96	7.178	8.03
3	54	51	54	0.3847	(0.081)	0.0428	(0.008)	0.108	(0.029)	8	5	10	4.861	9.728	8.07
4	32	32	31	0.132	(0.051)	0.06775	(0.019)	0.042	(0.011)	9	7	8	5.8394	4.5059	5.16
5	40	37	37	0.143	(0.025)	0.238	(0.023)	0.145	(0.021)	10	8	11	6.79	5.613	7.56
6	43	47	45	0.0711	(0.017)	0.2037	(0.022)	0.038	(0.009)	7	9	7	5.374	5.6799	3.43
7	44	47	46	0.2232	(0.043)	0.1508	(0.066)	0.105	(0.016)	7	7	3	3.719	5.841	4.47
8	62	47	ND	0.0407	(0.007)	0.0655	(0.011)	ND	ND	7	11	ND	11.276	11.047	ND
9	48	48	45	0.1917	(0.045)	0.1622	(0.018)	0.199	(0.047)	11	10	8	6.341	5.899	6.88
10	50	45	48	0.199	(0.021)	0.09	(0.013)	0.192	(0.022)	9	8	4	8.22	7.86	4.02

ND= MEPs not-differentiated from background EMG at 110% RMT for this session
#AS= number of active sites
RMT= Resting motor threshold; MEP=Motor evoked potential
